# 

**Published:** 1907-11-09

**Authors:** 


CONTENTS.

| | PAGE |
|---|---|
| **The Early Notification of Births Act** | 129 |
| **Wanted—A State Examination** | 130 |
| **Annotations—** | |
| The Infants' Hospital—Dangerous Prescriptions—Diet and Personal Idiosyncrasy | 131 |
| **Medical Opinion and Movement** | 132 |
| **Hospital Clinics—** | |
| Gonorrhœal Infection in the Female and its Treatment. By F. J. McCann, M.D., F.R.C.S.(Eng.), M.R.C.P. | 133 |
| Rheumatic Pericarditis and its Consequences. By J. Walter Carr, M.D., F.R.C.P. | 135 |
| **Points in Treatment—** | |
| The Treatment of Bacteriuria | 137 |
| **Points in Surgery—** | |
| The Treatment of Fractures from a Common-sense Point of View | 139 |
| Colostomy | 140 |
| **Laryngology and Rhinology—** | |
| The Treatment of Tuberculous Laryngitis | 141 |
| **Practical Notes on Diagnosis and Treatment** | 142 |
| **Otology—** | |
| Concerning Operations for Otorrhœa | 143 |
| **Dermatology—** | |
| Some Illustrative Cases | 144 |
| **Therapeutics—** | |
| Prescriptions and Dispensing | 145 |
| **Resident Medical Officers' Department—** | |
| Anæsthetic Cases | 146 |
| **Hospital Administration—** | |
| The Inebriates Acts, 1897-1900 | 147 |
| Social Problems at the Church Congress | 148 |
| The Caird Cancer Hospital, Dundee Royal Infirmary | 149 |
| News and Coming Events | 150 |
| The Hospital Officers' Association | 151 |
| Story of the Insane from Year to Year | 151 |
| **Nursing Administration—** | |
| Religious Equality in Institutions | 152 |
| The Common Task | 153 |
| **Editor's Letter-Box—** | |
| "Professional Freedom" | 153 |
| Hypnotism in its Relation to Hysteria and to Psychasthenia | 154 |

